# Using a Deep Learning Method and Data from Two-Dimensional (2D) Marker-Less Video-Based Images for Walking Speed Classification

**DOI:** 10.3390/s21082836

**Published:** 2021-04-17

**Authors:** Tasriva Sikandar, Mohammad F. Rabbi, Kamarul H. Ghazali, Omar Altwijri, Mahdi Alqahtani, Mohammed Almijalli, Saleh Altayyar, Nizam U. Ahamed

**Affiliations:** 1 Faculty of Electrical and Electronics Engineering, Universiti Malaysia Pahang, Pekan 26600, Malaysia; tasrivasikandar@gmail.com (T.S.); kamarul@ump.edu.my (K.H.G.); 2 School of Allied Health Sciences, Griffith University, Gold Coast, QLD 4222, Australia; fazle.rabbi@griffithuni.edu.au; 3 Biomedical Technology Department, College of Applied Medical Sciences, King Saud University, Riyadh 11451, Saudi Arabia; oaltwijri@ksu.edu.sa (O.A.); amahdi@ksu.edu.sa (M.A.); malmijalli@ksu.edu.sa (M.A.); stayyar@ksu.edu.sa (S.A.); 4 Neuromuscular Research Laboratory/Warrior Human Performance Research Center, Department of Sports Medicine and Nutrition, University of Pittsburgh, Pittsburgh, PA 15203, USA

**Keywords:** 2D image, marker-less video, walking speed pattern, walking speed classification, quasi-periodic pattern, LSTM, deep learning, rehabilitation, human mobility, gait impairment

## Abstract

Human body measurement data related to walking can characterize functional movement and thereby become an important tool for health assessment. Single-camera-captured two-dimensional (2D) image sequences of marker-less walking individuals might be a simple approach for estimating human body measurement data which could be used in walking speed-related health assessment. Conventional body measurement data of 2D images are dependent on body-worn garments (used as segmental markers) and are susceptible to changes in the distance between the participant and camera in indoor and outdoor settings. In this study, we propose five ratio-based body measurement data that can be extracted from 2D images and can be used to classify three walking speeds (i.e., slow, normal, and fast) using a deep learning-based bidirectional long short-term memory classification model. The results showed that average classification accuracies of 88.08% and 79.18% could be achieved in indoor and outdoor environments, respectively. Additionally, the proposed ratio-based body measurement data are independent of body-worn garments and not susceptible to changes in the distance between the walking individual and camera. As a simple but efficient technique, the proposed walking speed classification has great potential to be employed in clinics and aged care homes.

## 1. Introduction

Walking ability is an important consideration during routine therapy treatment and rehabilitation following surgery and is crucial for human mobility, which enables predictions of quality of life, mortality, and morbidity [1,2]. Walking speed is a simple, rapid, and easily obtained assessment tool [3], but significantly affects all gait parameters, such as cadence, stride length, stance, and swing durations [4,5]. For a long time, walking speed has been used as an independent screening indicator of demographic characteristics (e.g., age and sex), functional activities (e.g., kinematic and kinetic patterns and spatiotemporal parameters), and various physical outcomes (e.g., activity-related fear of falling) in normal controlled individuals (e.g., healthy) and patients (e.g., Parkinson’s disease and osteoarthritis) [6,7,8,9,10]. Additionally, the functional movement performance of individuals with neuromuscular conditions, such as post-stroke and cerebral palsy, can be assessed based on their walking speed, which might have an impact on gait [9,10]. The gait speed of an individual with a physical impairment might be affected by changes in walking conditions, which do not appear to affect the gait speed of healthy individuals. For example, at similar walking speeds, patients with diseases such as Alzheimer’s disease exhibit a slower walking gait speed than healthy controls, and this difference might be a good indicator for classifying patients and healthy controls [11]. Furthermore, a slow walking speed in elderly individuals (>60 years) predicts increased morbidity and mortality [12]. Walking speed provides a significant contribution to health assessment, including changes in spatiotemporal, kinematic, and kinetic parameters during the gait cycle [13]. Therefore, the efficient classification of walking speed could play a vital role in the scrutinization of normal and abnormal gait measurements, particularly in gait-based assessments during a rehabilitation process, and might thus help improve clinical care and our understanding of gait balance.

Spatiotemporal gait data (e.g., walking speed, swing phase time, and double stance time) are the second most often used parameters, among two other parameters, namely, kinematic and kinetic walking gait parameters [14]. Spatiotemporal gait data are multidimensional time-domain sequences representing the evolution of body posture during a gait cycle [15]. Additionally, human gait is a form of cyclic motion regardless of the walking speed, and as a consequence, the time-domain sequences estimated from this motion contain periodic and/or quasi-periodic patterns [16]. Collected sequential spatiotemporal gait data are used in gait assessments where the periodic and/or quasi-periodic patterns are classified as normal (typical) or anomalous (atypical) gait in different neuromuscular conditions [10,17]. Typically, sequential spatiotemporal gait data from a walking individual are collected by optoelectronic motion capture systems using reflective marker-based (attached to the individual’s body) and/or marker-less approaches [18]. These approaches for gait recognition mostly rely on two-dimensional (2D) and three-dimensional (3D) gait analysis methods. Both the marker-less or marker-based approaches can be applied independently or in combination and can be widely used for gait measurement using 2D and 3D video systems, but marker-less technologies have more potential than marker-based approaches due to their advantages regarding cost, time, and need for highly skilled operators. In addition, although 3D, marker-based and/or marker-less techniques are well known for the analysis of walking gait [19,20], 3D approaches have many drawbacks, such as the need for multiple cameras with high image resolution, which usually then results in a longer computational time, specific repeated calibration procedures, a complex process for time synchronization between cameras, and the need for a large space to record gait data [21]. Therefore, a 2D technique with a less complicated camera setup (such as a single camera) is an alternative approach for the efficient assessment of walking gait. Notably, any sequential spatiotemporal gait data can also be estimated from a single-camera-based marker-less 2D video system employing lateral-view video of a walking individual because continuous 2D image sequences from the video can show the continuous body postures of human gait [21,22]. This 2D approach is currently gaining popularity as an alternative to the marker-based optoelectronic system due to its simplicity, rapidity, and ability to potentially provide more significant assessments of human movement in research and clinical practice [23,24,25,26].

Several research studies have investigated walking gait (particularly speed-related parameters) using a 2D setup. For example, Castelli et al. estimated three types of walking speed (i.e., slow, comfortable, and fast) using body measurement data from walking individuals, such as the unilateral joint kinematics of the individual’s hip, knee, ankle, and pelvic tilt [21]. However, their extracted body measurement data highly depended on the garments worn by the walking individuals (i.e., socks and undergarments), which were used as segmental markers for tracking foot and pelvis parameters in the image [21]. A study conducted by Verlekar et al. estimated walking speed using the lower-body width of the walking individuals [22], but a walking individual’s body measurement data, such as height, mid-body width, lower-body width, and body area, in an image show inconsistent variations depending on the distance between the individual and the camera in different environments (e.g., indoor and outdoor settings) [27]. Thus, the results show that body measurement data that depend on the distance between the walking individual and camera might produce varying walking speed patterns for the same individual due to the camera configuration [22]. One possible solution for this limitation could be to scale or resize the image sequences of the video to normalize the walking individual’s body measurements in each image, but this process might cause visual distortion and degrade the image quality due to squeezing or stretching [28]. Another possible solution for this limitation could be to use the walking individual-to-camera distance independent body measurement data, which would produce stable walking speed patterns [27]. A study by Zeng and Wang proposed ratio-based data (such as body height–width ratio data), which are stable regardless of the distance between the walking individual and the camera [27]. In addition to body height–width ratio data, the study [27] also utilized inconsistent body measurements, such as the mid-body width, lower-body width, and body area data, to establish the walking speed pattern used for walking speed classification. The above-described studies indicate a further need for establishing ratio-based body measurement data that (a) can be extracted from 2D image sequences without the use of any marker, (b) are consistent regardless of the distance between the participant and camera in both indoor and outdoor environments, and (c) exhibit consistent periodic (or quasi-periodic) walking patterns suitable for walking speed classification. However, to our knowledge, this walking gait-related classification task has not been directly investigated using any computational intelligence methods.

Artificial intelligence (AI) techniques such as machine learning and deep artificial neural network methods successfully applied and provided new predictive models for complex gait analysis [29,30]. Therefore, a good classification method is needed for the classification of any gait-related task (e.g., walking speed patterns) with reliable and good accuracy [15]. Among the published studies on walking speed estimated from lateral-view 2D images of marker-less walking individuals [21,22,27], only that conducted by Zeng et al. directly investigated an individual’s walking speed classification; these researchers employed the radial basis function (RBF) neural network to solve the classification task [27]. More recently, Khokhlova et al. reported a strongly predictive performance model with a large capacity to learn, the ability to capture long-term temporal dependencies, and the capacity to use variable-length observations that was developed based on the recurrent neural network (RNN)-based deep learning (DL) method long short-term memory (LSTM) for sequential data classification [15]. Additionally, some other image-related classification tasks, such as handwriting recognition [31], speech recognition [32], and text classification [33], have been performed using LSTM and its successor methods (e.g., bidirectional LSTM (biLSTM) and convolution neural network LSTM (CNN-LSTM)). In support of this, LSTM approaches are also currently gaining popularity for clinical gait classification tasks, such as pathological [15] and impairment gait classification [34], due to their promising applicability in labeling sequential gait data. Furthermore, previous research studies have shown that biLSTM exhibits better classification accuracy than LSTM [35]. In general, both the biLSTM and LSTM DL methods need large datasets for training and validation purposes to obtain good accuracy and to avoid data overfitting and poor generalization [36,37]. However, there is lack of availability of sources (i.e., databases) providing a large clinical gait dataset, particularly of lateral-view 2D images of marker-less individuals walking over different ranges of controlled walking speed trials [38,39]. More specifically, there is a limited number of datasets consisting of a small number of subjects with lateral-view image sequences, few variations among controlled walking speed trials, and data collected in limited environments (e.g., either indoor or outdoor settings) that exhibit restricted licensing for public use [21,40]. To overcome this complexity, our study used large gait-related datasets from two publicly available state-of-the-art databases, namely, the Osaka University—Institute of Scientific and Industrial research (OU-ISIR) dataset A [41], and the Institute of Automation at the Chinese Academy of Sciences (CASIA) dataset C [42]. These publicly available image datasets from these two databases were recorded in large populations using lateral-view videos of walking individuals obtained using a single 2D camera (marker-less) and exhibit substantially varied controlled walking speed trials. The gait data from OU-ISIR dataset A and CASIA dataset C were obtained in indoor (treadmill) and outdoor (overground) settings, respectively. A number of previous studies have used these two datasets for vision-based gait recognition and obtained a reliable performance [43,44,45]. One prominent study by Verlekar et al. [46] suggested that images from both datasets could be a possible solution for studies on walking speed pattern recognition that need a large population dataset of lateral-view 2D images of marker-less walking individuals. However, to our knowledge, walking speed patterns have not been previously classified using these datasets and state-of-the-art computational intelligence techniques, such as the biLSTM DL algorithm, to obtain the most reliable and highest accuracy.

The aim of this study was to investigate potential ratio-based body measurement data that (a) can be extracted from lateral-view 2D image sequences without any marker, (b) are consistent with respect to the distance between the participant and camera in both indoor and outdoor settings, and (c) exhibit consistent quasi-periodic walking patterns that are suitable for walking speed classification. Additionally, this study aimed to investigate whether the walking speed patterns obtained from ratio-based body measurement data could be utilized to classify walking patterns in terms of speed using the DL model and thereby obtain reliable accuracy. To achieve these aims, this study proposed five ratio-based body measures: (i) the ratio of the full-body height to full-body width, (ii) the ratio of the full-body height to the mid-body width, (iii) the ratio of the full-body height to the lower-body width, (iv) the ratio of the apparent to the full-body area, and (v) the ratio of the area between two legs to the full-body area. This study hypothesized that these proposed five ratio-based body measurements exhibit the above-detailed qualities. Additionally, these five ratio-based body measurement data could be used to classify an individual’s walking speed pattern based on three speeds—slow, normal, and fast—by adopting the biLSTM model with a mean classification accuracy greater than 80% in indoor settings (using a treadmill, i.e., OU-ISIR dataset A) and greater than 75% in outdoor settings (overground, i.e., CASIA dataset C).

## 2. Methods

### 2.1. Participants and Datasets

In this study, 2D marker-less motion image sequences in the lateral view from 187 participants were considered to classify the walking speed patterns at three speeds: slow, normal, and fast. These image sequences were obtained from OU-ISIR dataset A [41] (obtained using an indoor treadmill) and CASIA dataset C [42] (obtained in outdoor overground settings) and separated to obtain our own datasets based on the walking speed patterns, namely, Dataset 1 (indoor trials) and Dataset 2 (outdoor trials), respectively, for training and testing purposes [41,42]. Three walking speeds were categorized: slow (2 to 3 km/h), normal (4 to 5 km/h), and fast (6 to 7 km/h) [42,47,48]. With both datasets, a walking speed pattern was established using five quasi-periodic signals calculated from the minimum number of image sequences (i.e., frames) available for the three above-described speeds. First, OU-ISIR dataset A consists of image sequences with a walking speed between 2 and 7 km/h for 34 participants, and these data were separated into slow, normal, and fast. Twelve image sequences were available for each participant, and in total, these were 408 image sequences with varying length and a minimum sequence length of 240 frames. As a result, Dataset 1 contains 136 walking speed patterns calculated consistently from 240 frames for each of the three speeds. In contrast, CASIA dataset C contains two, four, and two image sequences for slow, normal, and fast walking, respectively, and these were captured from 153 participants. Overall, the dataset contains 1224 image sequences of varying length, and the shortest sequence length is 35 frames. As a result, Dataset 2 contains 306, 612, and 306 walking speed patterns calculated from 35 frames of each of the slow, normal, and fast walking speeds, respectively.

### 2.2. Data Extraction and Gait Speed Pattern Creation

The five ratio-based body measurements estimated from image sequences were the following: (i) ratio of the full-body height to the full-body width (HW1), (ii) ratio of the full-body height to the mid-body width (HW2), (iii) ratio of the full-body height to the lower-body width (HW3), (iv) ratio of the apparent to the full-body area (A1), and (v) ratio of the area between two legs to the full-body area (A2). Notably, we directly used the original lateral-view silhouette image sequences provided in OU-ISIR dataset A and CASIA dataset C.

**Figure 1:**
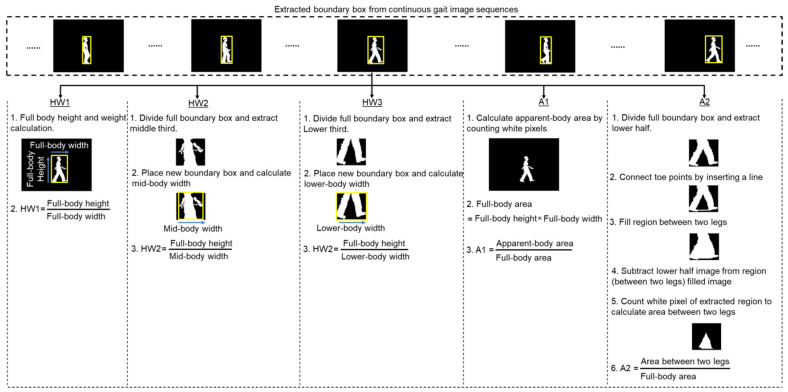
Graphical representation showing the extraction of the five ratio-based body measurements from an image sequence. Here, HW1—ratio of the full-body height to the full-body width; HW2—ratio of the full-body height to the mid-body width; HW3—ratio of the full-body height to the lower-body width; A1—ratio of the apparent to the full-body area; A2—ratio between area between legs and full-body area.

Figure 1 shows a graphical representation of the extraction of the five ratio-based body measurements obtained from an image sequence. To estimate the three height-to-width (i.e., HW1, HW2, and HW3) ratio-based body measurements, a rectangular boundary box was created around the whole body in each image using the *regionprops* function in MATLAB 2020a (MATLAB™, Natick, MA, USA). The height and width of the boundary box, which represent the full-body height and full-body width of the participant, respectively, were calculated from the properties of the function. We divided the full boundary box region into three equal parts and then placed a new rectangular boundary box around the object in the middle part to calculate the mid-body width and another rectangular boundary box around the object in the lower part to calculate the lower-body width. We then calculated the three height-to-width ratio-based body measurements using Equations (1)–(3).

(1) $$\mathrm{HW}1=\frac{\mathrm{Full-body}\;\mathrm{height}}{\mathrm{Full-body}\;\mathrm{width}}$$

(2) $$\mathrm{HW}2=\frac{\mathrm{Full-body}\;\mathrm{height}}{\mathrm{Mid-body}\;\mathrm{width}}$$

(3) $$\mathrm{HW}3=\frac{\mathrm{Full-body}\;\mathrm{height}}{\mathrm{Lower-body}\;\mathrm{width}}$$

To estimate the two area ratio-based body measurements, we calculated the participant’s apparent-body area in the image by counting the numbers of white pixels in the image. We also calculated the participant’s full-body area in the image by multiplying the full-body height and the full-body width. We divided the full boundary box region into two equal parts (upper and lower): the upper part extends from the head to the hip, and lower part extends from the hip to the leg. We removed any noise from the lower part of the image by deleting the smallest unconnected object to avoid even the smallest trace of a swinging hand. After connecting the toe points by inserting a line in the noise-free lower part of the image, the region between the two legs was filled using the *imfill* function in MATLAB 2020a (MATLAB™, Natick, MA, USA) and extracted by subtracting the noise-free lower part of the image from the image in which the region between the legs was filled. The area between the two legs was calculated by counting the number of white pixels in the extracted region between the two legs. We then calculated the two area-based body measurements using Equations (4) and (5).

(4) $$A1=\frac{\mathrm{Apparent-body}\;\mathrm{area}}{\mathrm{Full-body}\;\mathrm{area}}$$

(5) $$A2=\frac{\mathrm{Area}\;\mathrm{between}\;\mathrm{two}\;\mathrm{legs}}{\mathrm{Full-body}\;\mathrm{area}}$$

**Figure 2:**
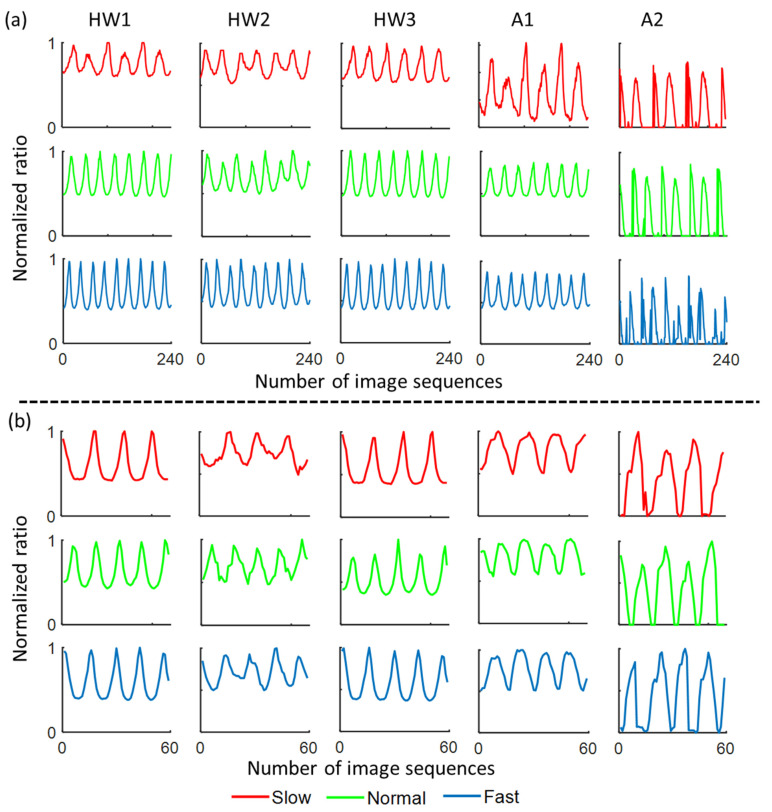
Quasi-periodic signals produced by the five ratio-based body measurements estimated from image sequences from one individual walking at three different speeds included in (**a**) OU-ISIR dataset A and (**b**) CASIA dataset C. Here, HW1—ratio of the full-body height to the full-body width; HW2—ratio of the full-body height to the mid-body width; HW3—ratio of the full-body height to the lower-body width; A1—ratio of the apparent to the full-body area; A2—ratio between area between legs and full-body area.

The variation in each of the five ratio-based body measurements over time produces quasi-periodic signals. All the quasi-periodic signals were normalized to 0 and 1 to eliminate the difference in the data obtained at the three speeds [27]. Figure 2 shows the five quasi-periodic signals calculated from image sequences in OU-ISIR dataset A (Figure 2a) and CASIA dataset C (Figure 2b) for a representative individual walking at three different speeds. After all quasi-periodic signals were obtained, the walking speed patterns were established to create Dataset 1 (indoor trials) and Dataset 2 (outdoor trials). To analyze the oscillatory behavior of the quasi-periodic signals produced by the five ratio-based body measurements (i.e., HW1, HW2, HW3, A1, and A2), we calculated the amplitude and frequency of the signals from a minimum sequence length of 240 and 35 frames of each signal in Dataset 1 and Dataset 2, respectively. The occurrence of local maxima in the quasi-periodic signals was calculated using the *findpeaks* function in MATLAB 2020a (MATLAB™, Natick, MA, USA) to estimate the frequency. Additionally, to compare the overall variation in the body measurements (such as full-body height, full-body width, mid-body width, lower-body width, apparent-body area, full-body area, and area between two legs) over consecutive frames at three speeds, we calculated the standard deviation (SD) from the mean over all image sequences.

### 2.3. Model Training and Cross-Validation

A biLSTM-based DL architecture was created based on the following five layers: an input layer of size five, a biLSTM layer with 100 hidden units, a fully connected layer with three outputs specifying the three classes, a softmax layer with an output between 0 and 1, and a classification layer with cross-entropy function for multi-class classification with three mutually exclusive classes [49,50,51]. The other properties of these layers were selected according to the default values in MATLAB 2020a (MATLAB™, Natick, MA, USA). The specified options for the training process are reported in Table 1. Previous research has shown that this simple setup is sufficient for obtaining non-overfitting and high-accuracy solutions to similar classification problems [52,53].

**Figure 3:**
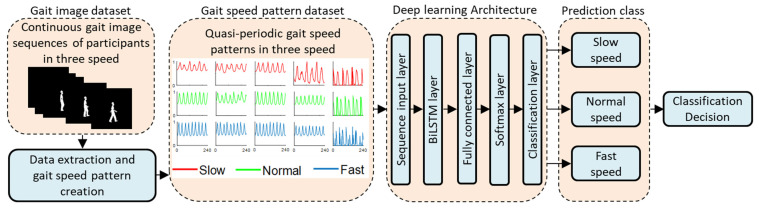
Workflow of the study.

To ensure that the classification approach was robust and that the data were not overfitted, the performance of the developed DL-based model was evaluated using two cross-validation methods: Method 1, which consisted of k-fold cross validation with training, validation, and testing subsamples, and Method 2, which consisted of repeated random sub-sampling cross-validation with training, validation, and testing subsamples [54]. In this study, both Dataset 1 and Dataset 2 can be considered multiclass datasets as they consist of three types of walking speed patterns. For Dataset 1, we applied 17-fold cross-validation with a total of 272 combinations of training, validation, and testing subsamples (Method 1) and repeated random sub-sampling cross-validation with 272 randomly selected training, validation, and testing subsamples (Method 2). For each fold or subsample in Methods 1 and 2, the training, testing, and validation data consisted of 88.24% (360 walking speed patterns), 5.88% (24 walking speed patterns), and 5.88% (24 walking speed patterns) of the walking speed patterns, respectively. For Dataset 2, we applied 18-fold cross-validation with a total of 306 combinations of training, validation, and testing subsamples (Method 1) and repeated random sub-sampling cross-validation with 306 randomly selected training, validation, and testing subsamples (Method 2). For each fold or subsample used in Methods 1 and 2, the training, testing, and validation data consisted of 88.9% (1088 walking speed patterns), 5.55% (68 walking speed patterns), and 5.55% (68 walking speed patterns) of the walking speed patterns, respectively. MATLAB 2020a (MATLAB™, Natick, MA, USA) software with an Intel(R) Core (TM) i5-2400CPU, 3.10 GHz computer was used for model training, validation, and testing the dataset. A complete workflow of the study is shown in Figure 3.

### 2.4. Statistical Analysis

To determine the differences in performance between the two cross-validation methods, SPSS statistical software (Version 25; IBM Corp., Armonk, NY, USA) was used to obtain basic descriptive statistics, such as the means (± standard deviations (SDs)), and to perform one-way repeated-measures analysis of variance (ANOVA) on all the classification accuracy results. The normalization of the data was assessed using the Shapiro–Wilk test (*p* > 0.05) prior to ANOVA, and Bonferroni adjustment was used for the post hoc analysis.

## 3. Results

The mean (± SD) amplitudes (in percentages %) and frequencies (number of maximum peaks per sequence) of the quasi-periodic signals produced by the five ratio-based body measurements at the three walking speeds are presented in Table 2 and Table 3, respectively. The results showed that a mean (± SD) amplitude between 51.66 (±7.33) and 80.50 (±0.99) was obtained using the three height-to-width ratio-based body measurements (HW1, HW2, and HW3) calculated from both datasets (Table 2). However, the area ratio-based body measurements (i.e., A1 and A2) yielded a mean (± SD) amplitude in the range of 9.53 (±2.16) to 58.71 (±0.74). The mean (± SD) frequency of the quasi-periodic signals from the five ratio-based body measurements showed trends similar to that found for the amplitude for both datasets (Table 3). In addition, the maximum and minimum frequencies obtained for the height-to-width ratio-based body measurements were 8.18 (±0.65) and 2.64 (±0.45), respectively, and those found for the area ratio-based body measurements were 8.10 (±0.65) and 2.47 (±0.58), respectively.

The overall variation in the body measurements (such as the full-body height, full-body width, mid-body width, lower-body width, apparent body area, full-body area, and area between the legs) over consecutive frames at the three speeds was calculated using the standard deviation (SD) from the mean over all image sequences and is presented (in terms of percentages, %) in Table 4. Minor variation was found in the participants’ body height with both datasets (Table 4): the minimum variation was ±0.50, and maximum variation was ±2.52. In contrast, substantial variation was found in the widths (minimum variation of ±9.65 and maximum variation of ±20.91) and areas (minimum variation of ±5.23 and maximum variation of ±30.45) of the body over time with both datasets.

The mean (± SD) classification accuracy of the experimental model was found to equal 88.05 (±8.85)% and 88.08 (±8.77)% using Methods 1 and 2, respectively (Table 5), with Dataset 1 (indoor trials), whereas mean (± SD) classification accuracies of 77.52 (±7.89)% and 79.18 (±9.51)% were achieved using Methods 1 and 2, respectively, with Dataset 2 (outdoor trials). Further descriptive statistics of the classification accuracies obtained with the training, validation, and testing data generated using the two cross-validation methods with the two datasets are provided in Table 5. The ANOVA results showed no significant differences (*p* > 0.05) in the overall classification accuracies obtained with Dataset 1 between the two methods. Additionally, no significant differences (*p* > 0.05) in the overall classification accuracies were found between the two methods with Dataset 2. Average time (min) for model training was 17.43 and 17.85 min for Method 1 and Method 2, respectively, using Dataset 1, while the time was 9.71 and 10.20 min for the two respective models when using Dataset 2.

## 4. Discussion

The main goal of the study was to investigate ratio-based body measurement data that can be extracted from marker-less 2D image sequences and are independent of the distance between the camera and the walking participant. Additionally, this study assessed whether these ratio-based body measurement data could be reliably and accurately utilized to classify an individual’s walking patterns in terms of speed in both indoor (treadmill trial) and outdoor (overground trial) environments using the biLSTM DL model.

This study constitutes the first comprehensive analysis of walking gait speed patterns using five ratio-based body measurements from 2D video images: three body measurements were calculated based on the ratio of the body height to width (HW1, HW2, and HW3), and the other two body measurements were based on ratios of body areas (i.e., A1 and A2). All five ratio-based body measurements showed a quasi-periodic nature over time in image sequences captured in both indoor (treadmill trial) and outdoor (overground trial) environments. The results proved that the overall amplitude of the quasi-periodic signals obtained with the ratio-based body measurements decreased with an increase in the walking speed, and this finding was obtained with both Dataset 1 and Dataset 2 (Table 2). A reason for this result is that regardless of the walking speed, only a minor variation was found in the participants’ body height, whereas significant variation was found in the widths and areas of the body over time (Table 4) [27,55]. More specifically, the widths and areas of body decreased to minimum values when the legs were together and both hands were straight along the body during the early stance and mid-swing phases of the gait cycle. Subsequently, these widths and areas reached maximum values when the legs and hands were furthest apart in opposite directions during the late-stance and late-swing phases of the gait cycle. The swinging of hands and legs in opposite directions increases the widths and areas of the body as the walking speed is increased. As a result, the variation in these widths and areas increased as the walking speed increased (Table 4). Therefore, the average amplitude of the quasi-periodic signals obtained with the three height–width ratio-based body measurements (HW1, HW2, and HW3) decreased as the walking speed increased. However, a slightly different variation in the amplitude was obtained with the area ratio-based body measurements (A1 and A2). The above explanations are supported by the results from previous studies, which also showed that the amplitudes of the cadence, step length, stride length, and stance duration are decreased at slower speeds and increased at faster speeds [56,57]. Again, in contrast to the amplitude, the average frequency of the quasi-periodic signals obtained with all five parameters increases proportionally with the speed when Dataset 1 was used because the swinging of both the upper and lower limbs is greater at faster walking speeds (Table 3). This explanation is supported by previous studies, which suggested that the hand swing frequency, step frequency, and stride frequency increase with increases in the walking speed and that the hand swing gradually changes from synchronous with the step frequency to locking into the stride frequency [58,59]. Note that the frequency of the ratio-based body measurements estimated using the image sequences in Dataset 2 did not follow the same trend as those obtained with Dataset 1, and this difference could be due to the smaller number of image sequences obtained in an outdoor environment and thus a smaller number of data points [21,40]. Both the amplitude and frequency of all ratio-based body measurements exhibited variation over the image sequences, and therefore, the ratio-based body measurements could be used to classify the walking patterns at different speeds. Our proposed five ratio-based measurements are more appropriate for indoor environments when compared to outdoor environments. However, the potential of the proposed measurements indicates further investigation for use in outdoor environments.

The experimental DL-based model achieved mean classification accuracies of 88.05% and 88.08% using cross-validation Methods 1 and 2 on Dataset 1, respectively (mean accuracy, Table 5). Although the overall classification accuracies obtained using cross-validation Methods 1 and 2 and on Dataset 1 ranged from 41.67% to 100% and from 37.50% to 100%, respectively, almost 50% of the trained models achieved classification accuracies higher than 89%, as demonstrated by applying both cross-validation methods with Dataset 1 (min–max accuracy and 50th percentile accuracy, Table 5). Only a few models compared with the total number of trained and tested models achieved low classification accuracies (number of outliers, Table 5). The model tested using Dataset 2 achieved mean classification accuracies of 77.52% and 79.18% using Methods 1 and 2, respectively (mean accuracy, Table 5). Although the classification accuracies obtained using both methods ranged from 25% to 100% with Dataset 2, almost 50% of the trained models achieved a classification accuracy with Dataset 2 greater than 75% with both methods (min–max accuracy and 50th percentile accuracy, Table 5). Some models achieved low classification accuracies, but this amount is small compared with the total number of trained and tested models (number of outliers, Table 5). The above findings are rational because Dataset 1 was created using images acquired in a controlled indoor treadmill trial environment, whereas Dataset 2 was established using images from an outdoor field trial with a more challenging environment [60]. Additionally, the current study achieved an excellent classification result, but the results are slightly different compared with those obtained in a previous study [27] on walking speed classification due to the cross-validation methods used in both studies. More specifically, the previous study [27] trained the model with a multiclass setting, i.e., all three types of walking speed patterns, and tested the models using a single-class setting, i.e., any one of the three walking speed patterns, whereas the current study used a multiclass setting as well as multiple runs for the training, validation, and testing of the model, which is beneficial for achieving accurate classification accuracy and building a successful model [61,62].

The ratio-based body measurements used for walking speed classification in this study were successfully estimated from lateral-view 2D image sequences of marker-less walking individuals captured with a digital camera. The concept of estimating body measurements from lateral-view 2D image sequences of marker-less walking individuals captured with a digital camera is supported by previous studies [21,46]. However, the ratio-based body measurements used in the current study are more robust than those used in previous studies because they are independent of the use of a body-worn garment as a segmental marker and of variations in the distance between the walking individual and the camera. To examine whether the ratio-based body measurements are independent of variations in the distance between the walking individual and camera, two datasets, namely, OU-ISIR dataset A and CASIA dataset C, which include data from both indoor and outdoor environments and different participant–camera distance settings, were used in this study. Additionally, the extraction of the proposed ratio-based body measurements preserves the natural movement of the participants during data collection in an outdoor environment [23]. The ability of classifying the walking speed in an indoor environment with high classification accuracy and in an outdoor environment with moderate classification accuracy will enable clinicians to use this method for regular diagnosis in clinical settings and for gait monitoring in aged care homes [63].

Although the proposed method has great potential for use in regular diagnosis in clinical settings and gait monitoring, the method has only been tested with healthy participants. A population with gait impairment could not be assessed in this study due to the scarcity of substantially large datasets available in the current research community [38,39]. This issue will be taken into consideration in the future by creating a large low-resolution image-based dataset focusing on a range of walking speeds. Additionally, this study only classified walking speeds using height-to-width ratio-based and area-based body measurements. In the future, this study will be extended to estimate other spatiotemporal parameters, such as the stride length, step length, joint angles, joint angle velocity, and acceleration, such that we can obtain greater insights on the participants’ health and classify normal and abnormal gait patterns. Although in this study we have used silhouette-based analysis [22,46], we will extend the work to advanced feature extraction techniques, such as pose estimation techniques [64,65,66], in the future so that the classification can be done with real-time video. Furthermore, this study was conducted using the minimum sequence length for walking speed patterns. As a consequence, the sequence length was short in the outdoor dataset. In the future, this study will be extended to apply a maximum sequence length by bridging time lags to increase the sequence length, so that a more appropriate analysis can be done in outdoor settings. Finally, this study uses only the biLSTM method to conduct classification tasks. Other state-of-the-art classification algorithms will be applied in the future to obtain solutions for optimum classification accuracy.

## 5. Conclusions

In summary, our proposed ratio-based body measurements were successfully extracted from marker-less 2D image sequences without the need for any body-worn garments and did not show any variations due to changes in the distance between the walking individual and the camera. Additionally, our deep learning classification model showed excellent mean classification accuracies (88.08% and 79.18%) using a large dataset of lateral-view 2D images of marker-less walking individuals undergoing controlled walking trials at different speed ranges in both indoor (treadmill trial) and outdoor (overground trial) environments, respectively. The excellent results obtained in this study support the use of simple ratio-based body measurement data that evolve with changes in the walking speeds, produce periodic or quasi-periodic patterns, and, more importantly, can be estimated from marker-less digital camera images in the sagittal plane to classify walking speeds using the currently available deep learning method. As a simple but efficient technique, the proposed walking speed classification method has great potential to be used in clinical settings and aged care homes.

## Figures and Tables

**Table 1:** Options for the training process used for cross-validation.

Options	Settings
Weight optimization method	Adaptive moment estimation optimizer
The initial learning rate	0.001
Decay rate of squared gradient moving average	0.99
Gradient threshold method	‘global-12norm’
Gradient threshold	0.9
Maximum epochs	200
Size of the mini-batch for each training iteration	27
Data shuffling	‘never’
Validation frequency	22

**Table 2:** Average amplitude (in terms of percentages, %) of the quasi-periodic signals obtained with the five ratio-based body measurements.

Dataset	Speed	HW1	HW2	HW3	A1	A2
Dataset 1	Slow walk	69.07 (±0.99)	80.50 (±0.99)	61.10 (±1.08)	55.72 (±0.74)	19.53 (±2.20)
	Normal walk	63.62 (±0.98)	71.78 (±0.86)	60.31 (±1.21)	58.71 (±0.74)	25.96 (±2.19)
	Fast walk	57.09 (±2.00)	64.58 (±1.79)	56.67 (±2.08)	56.38 (±1.51)	22.57 (±2.38)
Dataset 2	Slow walk	60.43 (±4.77)	71.85 (±2.91)	54.86 (±4.81)	46.40 (±2.36)	11.11 (±2.01)
	Normal walk	57.73 (±6.42)	66.58 (±4.67)	53.60 (±6.60)	10.77 (±0.75)	4.21 (±0.78)
	Fast walk	55.15 (±7.17)	64.09 (±5.59)	51.66 (±7.33)	43.14 (±3.34)	9.53 (±2.16)

HW1—ratio of the full-body height to the full-body width; HW2—ratio of the full-body height to the mid-body width; HW3—ratio of the full-body height to the lower-body width; A1—ratio of the apparent to the full-body area; A2—ratio of area between legs and full-body area.

**Table 3:** Average frequency (in terms of the number of maximum peaks per sequence) of the quasi-periodic signals obtained with the five ratio-based body measurements.

Dataset	Speed	HW1	HW2	HW3	A1	A2
Dataset 1	Slow walk	6.40 (±0.92)	6.15 (±0.78)	6.29 (±0.87)	7.03 (±1.00)	5.86 (±0.85)
	Normal walk	6.86 (±0.72)	6.93 (±0.66)	6.88 (±0.73)	7.06 (±0.64)	7.21 (±0.68)
	Fast walk	8.14 (±0.61)	7.60 (±1.02)	8.18 (±0.65)	8.10 (±0.65)	7.93 (±0.70)
Dataset 2	Slow walk	2.69 (±0.41)	2.76 (±0.42)	2.74 (±0.39)	3.31 (±0.45)	2.47 (±0.58)
	Normal walk	2.64 (±0.42)	2.64 (±0.45)	2.68 (±0.46)	2.97 (±0.45)	2.62 (±0.50)
	Fast walk	2.66 (±0.38)	2.76 (±0.36)	2.66 (±0.36)	2.88 (±0.42)	2.68 (±0.53)

HW1—ratio of the full-body height to the full-body width; HW2—ratio of the full-body height to the mid-body width; HW3—ratio of the full-body height to the lower-body width; A1—ratio of the apparent to the full-body area; A2—ratio of area between legs and full-body area.

**Table 4:** Variation in the body measurements over consecutive frames at three walking speeds. This variation was calculated using the standard deviation (SD) from the mean over all image sequences and is presented in terms of percentages (%).

Dataset	Speed	Full-Body Height	Full-Body Width	Mid-Body Width	Lower-Body Width	Apparent-Body Area	Full-Body Area	Area between Legs
Dataset 1	Slow	±0.50	±12.26	±9.65	±15.19	±5.23	±12.21	±27.97
	Normal	±0.70	±16.13	±13.47	±17.87	±6.44	±16.02	±30.45
	Fast	±0.92	±18.94	±16.65	±19.79	±7.16	±18.73	±29.51
Dataset 2	Slow	±2.40	±17.45	±12.95	±18.68	±9.74	±17.54	±22.75
	Normal	±2.20	±19.00	±15.12	±19.90	±10.26	±18.86	±24.12
	Fast	±2.52	±20.05	±16.42	±20.91	±10.50	±19.90	±24.95

**Table 5:** Descriptive statistics of the classification accuracies obtained with the training, validation, and testing data and the two cross-validation methods with the two datasets.

Descriptive Statistics	Dataset 1 (Indoor Trials)		Dataset 2 (Outdoor Trials)	
	Method 1	Method 2	Method 1	Method 2
Number of cross-validation experiments performed	272	272	306	306
Mean (± SD) accuracy	88.05 (±8.85)%	88.08 (±8.77)%	77.52 (±7.89)%	79.18 (±9.51)%
25th percentile accuracy	83.33%	83.33%	75.00%	75.00%
50th percentile or median accuracy	89.58%	91.67%	75.00%	75.00%
75th percentile accuracy	95.83%	95.83%	76.47%	83.82%
Minimum accuracy	41.67%	37.50%	25.00%	25.00%
Maximum accuracy	100.00%	100.00%	100.00%	100.00%
Lower adjacent accuracy	66.67%	70.83%	73.53%	69.12%
Upper adjacent accuracy	100.00%	100.00%	77.94%	95.95%
Accuracy range	58.33%	62.50%	75.00%	75.00%
Interquartile accuracy range	12.50%	12.50%	1.47%	8.82%
Number of outliers	5	4	81	26
Average training time (min)	17.43	17.85	9.71	10.20

SD—standard deviation.

## Data Availability

The data generated and/or analyses for the current study are available from the following publicly available databases: 1. Osaka University—Institute of Scientific and Industrial research (OU-ISIR) Dataset A: (www.am.sanken.osaka-u.ac.jp/BiometricDB/GaitTM.html (accessed on 4 May 2020)). 2. The Institute of Automation, Chinese Academy of Sciences (CASIA) Dataset C: (www.cbsr.ia.ac.cn/english/Gait%20Databases.asp (accessed on 10 May 2020)).
